# Bilateral Pancreaticopleural Fistula Masquerading as Thoracic Disease in Chronic Calculous Pancreatitis

**DOI:** 10.3390/diagnostics16050720

**Published:** 2026-02-28

**Authors:** Helen Bolanaki, Francesk Mulita, Ioannis Tzimagiorgis, Ioannis Chrysafis, Hippocrates Moschouris, Nikolaos Courcoutsakis, Savas P. Deftereos, Anastasios J. Karayiannakis

**Affiliations:** 1 Second Department of Surgery, Medical School, Democritus University of Thrace, 68100 Alexandroupolis, Greece; bolanakie@gmail.com (H.B.); med5507@ac.upatras.gr (F.M.); tzimagiorgis@hotmail.com (I.T.); 2 Department of Surgery, Medical School, University of Patras, 26504 Patras, Greece; 3 Department of Radiology, Medical School, Democritus University of Thrace, 68100 Alexandroupolis, Greece; jchrysafis@hotmail.com (I.C.); imoschou@med.duth.gr (H.M.); ncourcou@med.duth.gr (N.C.); sdefter@med.duth.gr (S.P.D.)

**Keywords:** pancreaticopleural fistula, chronic pancreatitis, bilateral pleural effusion, pancreatic duct disruption, calculous pancreatitis, magnetic resonance cholangiopancreatography, distal pancreatectomy, internal pancreatic fistula

## Abstract

**Background**: Pancreaticopleural fistula is a rare complication of chronic pancreatitis resulting from pancreatic duct disruption, typically presenting with pleural effusion and predominant respiratory symptoms. Bilateral pleural involvement is exceptionally uncommon and poses significant diagnostic and therapeutic challenges. **Case Presentation**: A 56-year-old man with a history of chronic alcohol abuse presented with progressive dyspnea and mild epigastric pain. Imaging revealed bilateral pleural effusions, an atrophic pancreas with a markedly dilated main pancreatic duct containing calculi, and a fistulous tract extending from the pancreatic body through the esophageal hiatus into the mediastinum. Magnetic resonance cholangiopancreatography confirmed the diagnosis of chronic calculous pancreatitis complicated by a pancreaticopleural fistula. After unsuccessful conservative management, the patient underwent distal pancreatectomy, resection of the fistulous tract, and Roux-en-Y pancreatojejunostomy. The postoperative course was uneventful, with complete resolution of pleural effusions and sustained clinical improvement. **Conclusions**: This case highlights the importance of considering pancreaticopleural fistula in patients with unexplained pleural effusions and minimal abdominal symptoms, particularly in the context of chronic pancreatitis. Bilateral involvement, although rare, should not preclude timely diagnosis. Appropriate diagnostic studies by computed tomography, magnetic resonance imaging, and magnetic resonance cholangiopancreatography are crucial for establishing the diagnosis. Surgical management offers definitive treatment in patients with ductal obstruction and calculous disease, resulting in excellent long-term outcomes.

## 1. Introduction

Pancreaticopleural fistula (PPF) is a rare but well-recognized complication of pancreatic disease, first described in medical literature in the late 1960s [1,2]. Together with pancreatic ascites, it is classified as an internal pancreatic fistula, a group of conditions sharing a common pathophysiological mechanism involving disruption of the main pancreatic duct (PD) and leakage of enzyme-rich pancreatic secretions into adjacent body cavities [3,4]. Posterior ductal disruptions permit pancreatic fluid to dissect through the retroperitoneum, ascend into the mediastinum via the aortic or esophageal hiatus, and ultimately drain into one or both pleural cavities [5,6].

In contrast to pleural effusions associated with acute pancreatitis, pancreaticopleural fistula is typically not accompanied by active pancreatic inflammation. Instead, it most frequently occurs in the setting of chronic, relapsing pancreatitis, particularly alcohol-induced disease [4,7]. Affected patients often have a history of long-term alcohol consumption and previous episodes of pancreatitis. Less common etiologies include gallstone disease, idiopathic pancreatitis, pancreatic trauma, and congenital anomalies of the pancreatic ductal system, especially in pediatric patients [6,8].

Clinically, PPF presents a considerable diagnostic challenge due to the predominance of thoracic rather than abdominal symptoms. Patients commonly present with dyspnea, cough, or chest discomfort secondary to large, recurrent pleural effusions, while gastrointestinal symptoms such as abdominal pain, nausea, vomiting, or diarrhea may be mild or entirely absent [3,5]. As a result, the initial diagnostic evaluation often focuses on pulmonary or cardiac pathology, leading to delayed identification of the pancreatic origin of the effusion and prolonged morbidity [7,9].

Pancreatic fistulas are estimated to occur in approximately 0.4% of acute pancreatitis patients and in up to 4.5% of patients with pancreatic pseudocysts, with 70–90% of cases arising as a complication of chronic pancreatitis [3,6,10,11]. Bilateral pleural involvement is exceptionally uncommon, with only isolated cases reported in the literature, highlighting the rarity and clinical significance of such presentations [8,11,12,13]. Here, we present a patient with chronic calculous pancreatitis who presented with bilateral pleural effusions due to pancreaticopleural fistula that was successfully diagnosed by computed tomography, magnetic resonance imaging, and magnetic resonance cholangiopancreatography and managed with definitive surgical treatment.

## 2. Case Presentation

A 56-year-old man presented with progressive dyspnea over a two-month period, accompanied by mild epigastric pain. His medical history was otherwise unremarkable, with no reported recent abdominal trauma. He reported chronic alcohol abuse, consuming approximately 3 L of beer daily for the past 12 years, and heavy smoking (30–40 cigarettes per day) for 20 years.

On admission, the patient was afebrile with stable vital signs. Physical examination revealed decreased breath sounds and dullness to percussion over both lower lung fields. Abdominal examination showed mild epigastric tenderness without guarding, rebound tenderness, or distension.

Laboratory evaluation demonstrated mild anemia (hematocrit 31.8%, hemoglobin 11.8 g/dL), marked leukocytosis (30.92 K/μL with 93% neutrophils), and thrombocytosis (780 K/μL). Inflammatory markers were elevated, with a C-reactive protein level of 24.50 mg/L. Serum total protein (4.9 g/dL) and albumin (2.8 g/dL) were reduced, and hypocalcemia was noted (7.1 mg/dL). Serum amylase was markedly elevated at 799 U/L. Liver and renal function tests were within normal limits.

**Figure 1:**
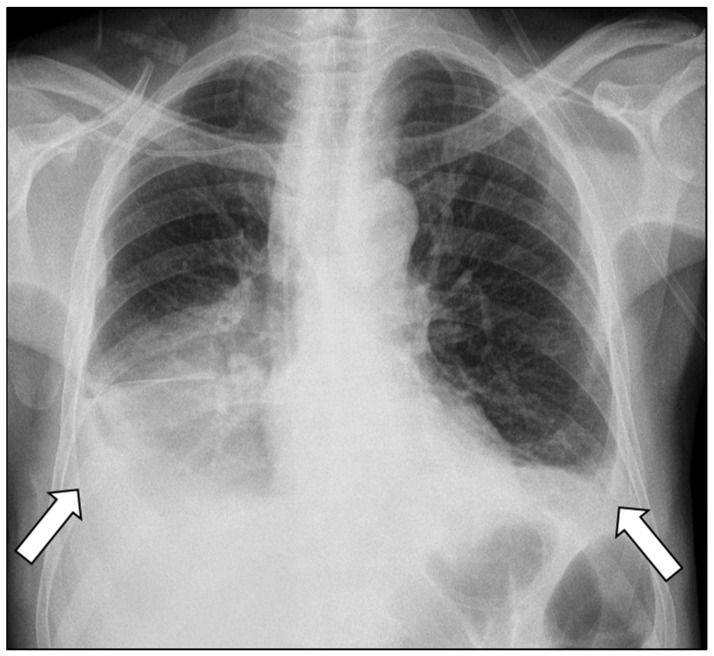
Chest radiograph demonstrating bilateral pleural effusions (arrows), more pronounced on the right side.

**Figure 2:**
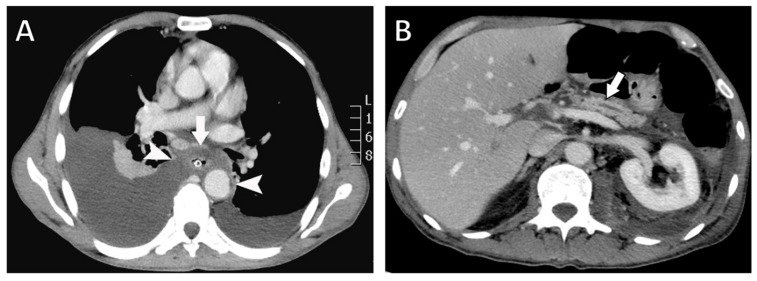
Contrast-enhanced computed tomography (CT) findings. (**A**) Axial CT image showing a fluid collection surrounding the esophagus (arrow) extending through the mediastinum and communicating with both pleural cavities (arrowheads). A nasogastric tube is visualized within the esophageal lumen. (**B**) Axial CT image demonstrating pancreatic atrophy with dilatation of the main pancreatic duct (arrow).

**Figure 3:**
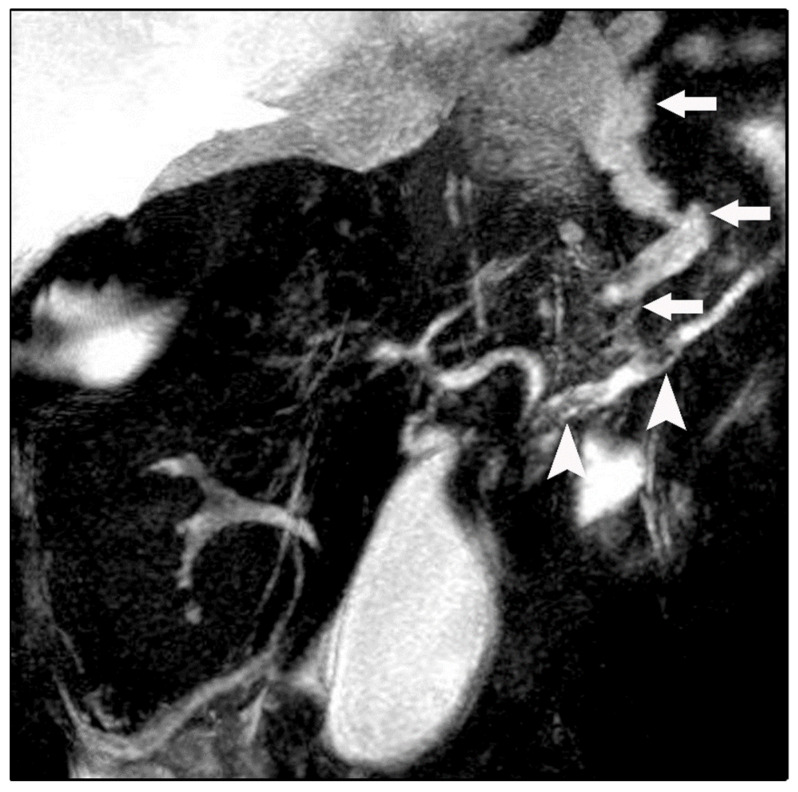
Magnetic resonance cholangiopancreatography (MRCP) revealing a fistulous fluid tract arising from a disruption of the main pancreatic duct and coursing toward the esophageal hiatus (arrows). Intraductal filling defects consistent with pancreatic duct calculi are also noted (arrowheads).

Chest radiography revealed bilateral pleural effusions, more pronounced on the right side (Figure 1). Contrast-enhanced thoracoabdominal computed tomography (CT) confirmed bilateral pleural effusions with associated ascites (Figure 2). The pancreas appeared atrophic with dilatation of the main pancreatic duct, consistent with chronic pancreatitis. A peripancreatic fluid collection extended toward the esophageal hiatus, coursing into the mediastinum and pleural spaces. Magnetic resonance imaging (MRI) confirmed pancreatic atrophy, while magnetic resonance cholangiopancreatography demonstrated an irregularly dilated main pancreatic duct measuring approximately 1 cm with intraductal filling defects. A fistulous tract originating from the pancreatic body and extending through the left side of the esophageal hiatus into the mediastinum was identified. The biliary tree and gallbladder were unremarkable, and no further pancreatic lesions were appreciated (Figure 3), thus not necessitating further evaluation with EUS. These findings established the diagnosis of chronic calculous pancreatitis complicated by a pancreaticopleural fistula. As imaging studies took place promptly following initial presentation, and the suspicion of pancreaticopleural fistula was established early, pleural fluid amylase levels were not sent for this patient.

Initial conservative management included bowel rest, total parenteral nutrition, and subcutaneous octreotide. After seven days of conservative treatment the pleural effusions did not improve. Endoscopic intervention and pancreatic duct stenting were initially considered during multidisciplinary evaluation. Given the marked pancreatic duct dilatation, multiple intraductal calculi and disruption of the main pancreatic duct at the pancreatic body, successful stenting and durable fistulous tract closure were deemed unlikely, in the setting of grossly disrupted PD anatomy and great stone burden. In addition, delaying definitive treatment carried the risk of worsening pleuropulmonary complications. Therefore, surgical treatment with resection of the fistulous tract and a distal pancreatectomy with a distal PJ anastomosis were deemed as the best option for definitive management that would ensure restored PD drainage. The disease course, potential complications, and therapeutic options were discussed with the patient and surgical management was elected.

**Figure 4:**
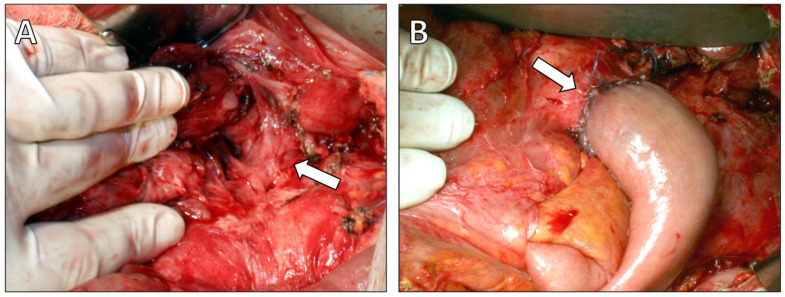
Intraoperative findings. (**A**) Intraoperative photograph demonstrating the fistulous tract entering the esophageal hiatus (arrow). (**B**) Intraoperative photograph showing the completed Roux-en-Y end-to-end pancreatojejunostomy following distal pancreatectomy (arrow).

At laparotomy, a moderate volume of free ascitic fluid was encountered and sampled for biochemical analysis, amylase measurement, and culture. Extensive inflammatory changes were noted in the peripancreatic region, lesser sac, and hepatoduodenal ligament, intraoperative findings consistent with chronic inflammatory process. A pancreatic duct disruption was identified in the pancreatic body, corresponding to the fistulous tract. Given the dilated pancreatic duct and intraductal calculi, resectional surgery was performed to relieve ductal obstruction. A distal pancreatectomy with splenectomy was carried out, the pancreatic duct was irrigated with saline, and stones were removed. The fistulous tract was dissected up to the esophageal hiatus and resected. Reconstruction was achieved by invaginating the pancreatic stump into a jejunal loop using a Roux-en-Y end-to-end pancreatojejunostomy, completed with a single-layer capsule-to-seromuscular anastomosis using interrupted absorbable sutures (Figure 4). A drain was placed posterior to the anastomosis, extending toward the esophageal hiatus. Ascitic fluid amylase measured 4380 U/L, and cultures were sterile.

The postoperative course was uneventful. Pleural effusions resolved gradually, and the patient was discharged on postoperative day 15. A postoperative ERCP was not deemed necessary as the patient had progressive resolution of his pleural effusions and no recurrent pancreatitis symptoms at short or long term follow up. At seven-year follow-up, he remains asymptomatic, with no recurrence of pleural effusions, ascites, or pancreatitis, although he continues to consume alcohol in reduced quantities.

## 3. Discussion

Pancreaticopleural fistula represents an uncommon but clinically significant manifestation of pancreatic duct disruption, most frequently arising in the context of chronic pancreatitis. The pathogenesis involves leakage of pancreatic secretions through a posterior ductal disruption, allowing fluid to traverse the retroperitoneum and ascend into the thoracic cavity, typically via the esophageal or aortic hiatus [5,14,15]. The fistulization dynamic in the setting of chronic pancreatitis can manifest in unusual presentations, including pancreato-mediastinal fistulas [16]. Fistulating disease such as these that stems from the pancreas can be often difficult to diagnose and treat sufficiently while avoiding recurrences. In the present case, imaging and intraoperative findings confirmed a ductal disruption at the level of the pancreatic body with extension of pancreatic fluid through the esophageal hiatus, consistent with this established mechanism. This pathophysiology explains the frequent absence of acute abdominal symptoms and the predominance of respiratory manifestations, which often leads to delayed diagnosis and extensive pulmonary evaluation before the pancreatic origin is recognized [3,16,17]. Indeed, our patient presented primarily with progressive dyspnea and only mild epigastric discomfort, illustrating this classic but misleading clinical pattern.

In most reported cases, pleural effusions associated with PPF are unilateral and left-sided, reflecting the anatomic proximity of the pancreatic tail to the left hemidiaphragm [18,19]. Bilateral pleural effusions, as observed in the present case, are exceedingly rare and have been described only sporadically in literature. The bilateral thoracic involvement in our patient likely reflects extensive mediastinal tracking of pancreatic fluid rather than separate fistulous tracts, suggesting an advanced and longstanding disease process [20,21]. The rarity of bilateral involvement further complicates diagnosis and underlines the importance of maintaining a high index of suspicion in patients with unexplained recurrent pleural effusions. Measurement of amylase level in the pleural effusion is crucial for the diagnosis of pancreaticopleural fistula with very high levels reported in the literature [13,17,21]. In our patient CT, MRI and MRCP were performed soon after his admission. The findings from imaging studies, particularly of MRCP, definitively established the underlying pathology and defined the fistulous tract. Therefore, measurement of amylase level in the pleural fluid was not considered necessary.

Imaging plays a central role in establishing the diagnosis of pancreaticopleural fistula. While computed tomography is useful for identifying pancreatic morphology, ductal dilatation, and associated fluid collections, it may fail to delineate the fistulous tract in a significant proportion of cases [21,22,23]. In our patient, CT imaging was instrumental in demonstrating mediastinal and pleural fluid extension but did not fully define the fistulous communication. Magnetic resonance cholangiopancreatography (MRCP) has emerged as the preferred noninvasive imaging modality, offering superior visualization of the pancreatic ductal anatomy and fistulous communication without the risks associated with invasive procedures [24,25]. MRCP in this case clearly demonstrated both the fistulous tract and intraductal calculi, directly influencing therapeutic decision-making. Endoscopic retrograde cholangiopancreatography (ERCP), although historically considered the diagnostic gold standard, is now primarily reserved for therapeutic intervention due to its invasiveness and risk of procedure-related pancreatitis [26].

Management of pancreaticopleural fistula remains controversial and must be individualized based on the anatomy and the degree of main pancreatic duct dilatation, the presence of ductal obstruction, and response to initial therapy. Conservative management—including bowel rest, total parenteral nutrition, and somatostatin analogues—may be successful in selected patients, particularly those with minimal ductal disruption and preserved ductal continuity [27,28]. Thoracocentesis may be required for symptomatic relief in patients with large or recurrent pleural effusions, but it does not address the underlying pancreatic duct disruption. Despite an initial trial of conservative therapy in our patient, the presence of marked ductal dilatation and calculous obstruction suggested a low likelihood of spontaneous fistula closure, a finding supported by previous studies [29].

Endoscopic intervention with pancreatic duct stenting has gained popularity as a minimally invasive therapeutic option aimed at reducing intraductal pressure and promoting fistula closure [30,31]. Nonetheless, endoscopic treatment may fail in the presence of complete ductal obstruction, extensive intraductal calculi, or distal ductal disruptions—features commonly encountered in chronic calculous pancreatitis [32]. In the present case, the combination of a dilated duct, multiple intraductal stones, and ductal disruption in the pancreatic body rendered endoscopic management suboptimal, favoring a primary surgical approach. In such cases, delayed surgical intervention may increase morbidity due to persistent leakage, infection, or pleural complications.

Surgical management remains the definitive treatment for pancreaticopleural fistula when conservative or endoscopic measures fail or are deemed unsuitable. Operative strategies are guided by ductal anatomy and include pancreatic resection, drainage procedures, or a combination thereof [33,34,35]. Distal pancreatectomy with splenectomy, as performed in the present case, is indicated in patients with ductal disruption involving the pancreatic body or tail, particularly when associated with intraductal stones and ductal dilatation [35,36]. Complete excision of the fistulous tract and reconstruction with Roux-en-Y pancreatojejunostomy ensured adequate pancreatic drainage and likely contributed to the durable outcome observed.

Long-term outcomes following appropriate surgical intervention are generally excellent, with high rates of fistula resolution and low recurrence when ductal pathology is adequately addressed [37]. The favorable seven-year follow-up in our patient, without recurrence of pleural effusions, ascites, or pancreatitis, supports the effectiveness of definitive surgical management in selected cases. Continued alcohol consumption, even at reduced levels, remains a concern and highlights the importance of long-term counselling and surveillance to prevent disease progression or new pancreatic complications [38,39,40,41].

## 4. Conclusions

Pancreaticopleural fistula is a rare and diagnostically challenging complication of chronic pancreatitis, often presenting with predominantly respiratory symptoms that may obscure the underlying pancreatic pathology.

The present report on this entity emphasizes the importance of thorough investigation of a patient with chronic pancreatitis who presents with ongoing dyspnea and large pleural effusions. Timely diagnosis is of paramount importance in providing definitive treatment. Pleural fluid amylase level is an effective diagnostic criterion, which ultimately needs supplementation with cross-sectional imaging modalities, particularly magnetic resonance cholangiopancreatography, to delineate the fistulous tract. Treatment of these cases in specialized HPB units with availability of endoscopic and advanced surgical treatment options is of paramount importance in achieving good outcomes.

## Data Availability

The data presented this study are available from the corresponding author upon request.
